# Sulfate turpentine: a resource of tick repellent compounds

**DOI:** 10.1007/s10493-017-0145-7

**Published:** 2017-06-06

**Authors:** Fredrik Schubert, Katinka Pålsson, Ellen Santangelo, Anna-Karin Borg-Karlson

**Affiliations:** Department of Chemistry, School of Chemical Science and Engineering, Ecological Chemistry Group, KTH, Royal Institute of Technology, 100 44 Stockholm, Sweden

**Keywords:** *Ixodes ricinus*, Repellency, Conifer turpentine, Borneol

## Abstract

Compounds with tick (*Ixodes ricinus*) repellent properties were isolated from sulfate turpentine consisting of Norway spruce (80%) and Scots pine (20%) from southern Sweden. The turpentine was divided into two fractions by distillation under reduced pressure resulting in one monoterpene hydrocarbon fraction and a residual containing higher boiling terpenoids. The monoterpene fraction was further oxidized with SeO_2_ to obtain oxygenated monoterpenes with potential tick repellent properties. The oxidized fraction and the high boiling distillation residual were each separated by medium pressure liquid chromatography. The fractions were tested for tick repellency and the compounds in those with highest tick repellency were identified by GC-MS. The fractions with highest repellency contained, mainly (−)-borneol, and mixtures of (+)- and (−)-1-terpineol and terpinen-4-ol. The enantiomers of borneol showed similar tick repellent properties.

## Introduction

Ticks have a worldwide distribution and are exclusively blood feeders. They are the most important vectors of disease agents in the northern hemisphere (Sonenshine 2003). *Lyme borreliosis* (Lyme disease) and tick-borne encephalitis (TBE) are two tick-borne infections of major importance. Both occur in Europe and Asia, with *Lyme borreliosis* also extending into North America (Sonenshine 2003). Each year it causes disease in 100,000–200,000 people in Europe. In Sweden alone 10,000 people become infected with Lyme borreliosis spirochetes (*Borrelia burgdorferi* s.l.) annually. Additionally, 5000–12,000 human cases of tick-borne encephalitis (TBE) occur in Europe, and in Sweden approximately 200–250 people become seriously ill with TBE (WHO 2014) annually. Other pathogens transmitted by *I. ricinus* to humans and domestic animals are the agents of anaplasmosis/ehrlichiosis (*Anaplasma* [formerly *Ehrlichia*] *phagocytophilum*], Q-fever [*Coxiella burnetii*], tularaemia [*Francisella tularensis*], and babesiosis [*Babesia divergens, Babesia microti*]) (Jaenson 1999).

Our aim for this study was to investigate if conifer turpentine from the pulping process (Södra Cell) could be used as a source for tick repellent compounds. Norway spruce (*Picea abies* L.) and Scots pine (*Pinus sylvestris* L.) are the two conifer species used in the pulping process in Sweden. The turpentine is obtained during the Kraft pulping process and is used as energy and/or for production of various solvents. There is an increasing interest in finding new “added values” for the turpentine and other side streams from the pulping processes. Turpentine is comprised of a large number of compounds many of which have the potential to be, or are proven to be active in biological systems. Certain compounds in the turpentine might protect conifer trees, against insects, or microorganisms such as bacteria and fungi. Turpentine could be a simple and easily available source of protection for humans and domestic animals against ticks is needed worldwide. Turpentine from the pulping process can be produced as a “green” and renewable natural source. Apart from its current primary use, as a fuel for lowering energy costs in the forest industry or as biofuel, we show here that parts of the turpentine have strong bioactive properties which could also be used as a tick repellent.

## Materials and methods

### Distillation and bioassay guided separation by MPLC

**Fig. 1:**
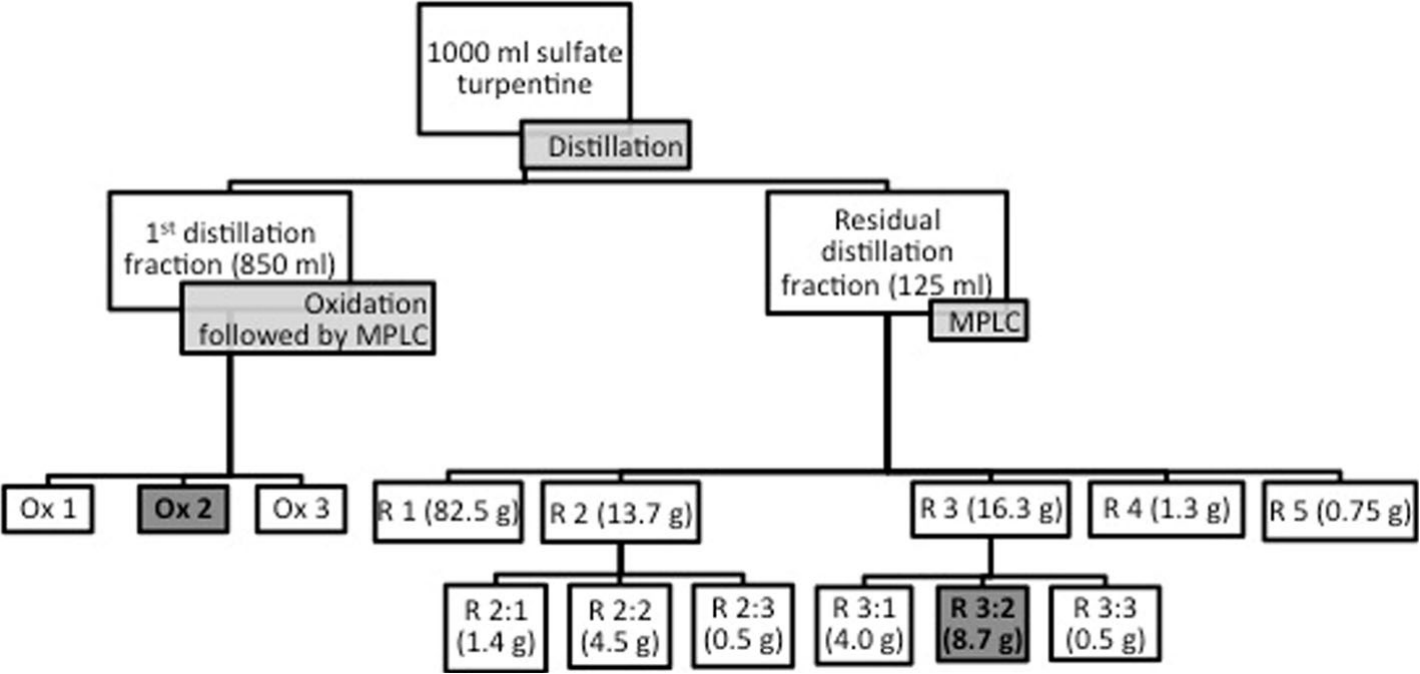
Distillation scheme of the turpentine

1000 ml sulfate turpentine from the wood of Norway spruce (~80%) and Scots pine (~20%) was obtained from Södra Cell Värö. The monoterpene hydrocarbon fraction of the sulfate turpentine was separated by distillation under reduced pressure. The remaining high boiling fraction containing mainly oxygenated mono- and sesquiterpenes (boiling above 150 °C at 10 psi; 125 ml) was separated by a medium pressure liquid chromatography system (MPLC, Bæckström Separo) described in Sofrata et al. (2011). The oil (125 ml) from the distillation was dissolved in 1000 ml hexane and pumped into a 50 mm column containing 30 cm of SiO_2_. Separation of the high boiling distillation fraction was made using a gradient of hexane/ethyl acetate. The eluated compounds were collected in 130 ml tubes and, based on TLC (thin layer chromatography) and Rf values, were pooled (Fig. 1) to five fractions: R1 (tubes 1–48), mainly hydrophobic, to fraction R5 (tubes 121–130), mainly hydrophilic. After showing tick repellent properties in a laboratory-screening test (see below), the medium polar fractions R2 (tubes 49–63) and R3 (tubes 64–84) were further separated into sub fractions and tested for tick repellent properties in a bioassay (Table 1).

### Oxidation of the monoterpene fraction

The initial fraction of the sulfate turpentine distillation (850 ml) was comprised mainly of monoterpene hydrocarbons. To enhance the commercial value of these large amounts of monoterpene hydrocarbons and to obtain oxygenated monoterpenes with tick repellent properties oxidation of the fraction was made using SeO_2_. In a 250 ml flask 11 g (0.1 mol) of SeO_2_, 150 ml of CH_2_ Cl_2_ and 50 ml of BuOOH (70%) were mixed. The monoterpene hydrocarbon fraction from the sulfate turpentine distillation was added over several minutes. The mixture was stirred for 24 h at 25 °C. Toluene was added and the CH_2_Cl_2_ was removed using a rotary evaporator. The product was further fractionated by MPLC using a gradient of hexane and ethyl acetate, as for the turpentine residual fractions. Three fractions (Ox 1–3) with increasing polarity (Fig. 1), according to TLC, were collected and tested for tick repellency.

### Repellency tests of *Ixodes ricinus* nymphs

#### Laboratory tests

Host-seeking (non-blood fed) nymphal *I. ricinus* were collected in a forested area in Stockholm, Sweden during May–September and kept at 80–90% relative humidity, 4 °C in complete darkness. Ticks used in the bioassays had been in captivity from 1 to 5 weeks and were discarded after one test. Prior to the test they were adapted to the test environment (22 °C, L: D 12:12 h) for 24 h.

A repellency bioassay on *I. ricinus* nymphs was carried out using the fractions of sulfate turpentine listed in Table 1. These bioassays were conducted, as described in Jaenson et al. (2006a, b) and Garboui et al. (2006), using one unfed *I. ricinus* nymph in a Falcon™ vial in each replicate. The Falcon vial is a 50 ml centrifugal tube, 116 × 29 mm, made of transparent plastic. The test fraction (100 µl of a 10% dilution in acetone) and the control substance (acetone) were applied with pipettes to separate cotton cloths. Each cloth was attached with a rubber band to the open upper end (7.0 cm^2^) of a Falcon vial. The vial walls were perforated with 12–15 small holes to prevent saturation of the air with the test substance, control substance or the scent of the experimenter. There were 20 replicates (n = 20) of each test fraction and control. A total of 100 µl of test substance was applied to a new cloth for each replicate. Each fraction was also tested using one cloth with 100 µl for 10 replicates (10% corresponding to 130 µg/cm^2^ and 1% to 13 µg/cm^2^, dilution in acetone). (±)-α-Terpineol and (±)-terpinen-4-ol, as well as the commercial borneol (comprising of 80% (−) and 20% (+) enantiomer) were tested in 5% dilution—corresponding to 65 µg/cm^2^ concentration (using 1 cloth for 10 replicates). In each replicate, half of the unfed nymphs were tested first with the control for 5 min, and then with the test substance for 5 min. The other half was tested with the test substance first, followed by the control. To simulate host stimuli, to attract the nymphs, the observer placed the palm of the hand on the outside surface of the cloth during the duration of the test. Ticks clinging to the cloth at 5 min after start of the test were recorded in the protocol as “attracted”, ticks that did not were recorded as “repelled”. Ticks that were below the 3 cm level of a 11.5 cm tall test tube (a Falcon™ vial) were regarded as “strongly repelled”.

### Further testing of borneol

Borneol (Fluka, 80% GC-purity 80% (−)- and 20% (+)-enantiomer), also containing 20% isoborneol; 95% pure (+)-borneol and 95% (−)-borneol (both Sigma Aldrich) were tested in 0.1% concentrations, as well as the mix of (+)-borneol and (−)-borneol (100 µl of either (+)- and (−)-borneol and 200 µl of the mix was applied to the cloth). (+)-Borneol and (−)-borneol were tested against each other to determine if there was any difference in repellency of the enantiomers (Fig. 4).

### Field trials

Two field experiments were conducted in June–July 2007 in the Stockholm area (N59º21.386’, E18º04.581’). In field trial I the most repellent MPLC-fraction of sulfate turpentine, single constituents and a commercially available MyggA^®^(containing 20% DEET (N,N-diethyltoluamide) were compared. In field trial II the most repellent MPLC fraction of sulfate turpentine and oxidation MPLC fractions were compared with MyggA^®^ (II).

### Field trial I comparing R3:2, borneol and MyggA^®^

The strongest tick repellent fraction (64–84: 56–78) R3:2, from the distilled sulfate turpentine, pure standard borneol (Fluka) and the commercially available repellent MyggA^®^ were tested under field conditions. A 1 m^2^ cloth was covered with 10 ml of the test substance or a control (acetone); substances were applied evenly to cover the whole downward surface of the cloth. The final concentrations of the test substances on the cloths were 65 mg/m^2^ (diluted in acetone) calculated based on the amount of test substance to cover the cloth surface (7.0 cm^2^) used in the lab test (5% dilution). Two cloths (one control, one containing the test substance) were pulled by two persons, parallel to each other, over the vegetation for 10 m, 15 times each day on 3 consecutive days for each substance. Every 10 m the cloths were turned over, each tick counted, removed and put into marked vials. The order in which the substances were tested was randomized. The cloths were stored separately in airtight heat resistant plastic bags (“Toppit Stekpåse” Melitta^®^) until the next day of testing.

### Field trial II comparing R3:2, Ox2 and MyggA^®^

In field trial II, fraction (64-84: 56–78) R3:2, the SeO_2_ oxidized terpene fraction (13–21) Ox 2 and the commercially available repellent MyggA^®^ were tested under field conditions in the same way as described in Field trial I. The concentrations of the test substances on the cloths were 130 mg/m^2.^

### Statistical analyses

Percentage repellency was calculated as [(no. of ticks recorded as attracted in the control − no. of ticks recorded as attracted in the test)/no. of ticks recorded as attracted in the control] × 100. For the data we also used the McNemar’s change test. Strong repellence was calculated as above, but individuals only had to move more than 30 mm to be considered as attracted. The Kolmogorov–Smirnov Two-Sample Test, using StatSoft STATISTICA 7.0 was performed to determine if the repellent effect of the enantiomers (+)-borneol and (−)-borneol were significantly different. The percentage repellency compared to the control was also calculated. For the field data we used the Kruskal–Wallis test for comparisons of nymph numbers between the substances tested and the control.

### Separation and identification of turpentine constituents

The active fractions were analyzed by gas chromatography-mass spectrometry (GC-MS). A Varian 3400 GC (Varian, Palo Alto, CA) connected to a Finnigan SSQ 7000 instrument with electronic ionization and the ion source at 150 °C. A DB–WAX fused silica capillary column (i.d. 0.25 mm, film thickness 0.25 µm and length 30 m) was used for the separation of the volatiles. The temperature programming was 40 °C (1 min) followed by 5 °C/min to 220 °C (12 min). The injector temperature was kept at 215 °C and helium was used as the carrier gas with a pressure of 67 kPa. The fractions and pure compounds were injected split less (1 µl) into the GC and identified by means of their retention times (GC–MS) and/or mass spectral data (MS) from authentic samples or referenced from the NIST (National Institute of Standards and Technology) library.

Selected chiral compounds were separated using a two dimensional GC instrument constituting two connected Varian 3400 GC:s the first with a DB-WAX column and the second a Cyclodextrin-β column (both Agilent; 30 m, ID 0.25 mm, film thickness 0.25 μm). For details see Borg-Karlson et al. (1993) and Persson et al. (1993).

## Results

The working scheme, including the various fractions obtained after distillation of the turpentine is presented in Fig. 1. Separation of the turpentine residual distillation fraction resulted in three main fractions (R1-5; R2:1–3 and R3:1–3) . The yields of each fraction are shown in Fig. 1. The most tick repellent fraction (R3:2; 8.7 g) obtained after two consecutive MPLC separations of approximately 1% of the chromatographic starting material. Additional oxidative treatment of the large monoterpene hydrocarbon fraction resulted in fractions Ox 1–3. The chemical characterization focused on the fractions with highest tick repellency.

### Laboratory tick repellency test

**Fig. 2:**
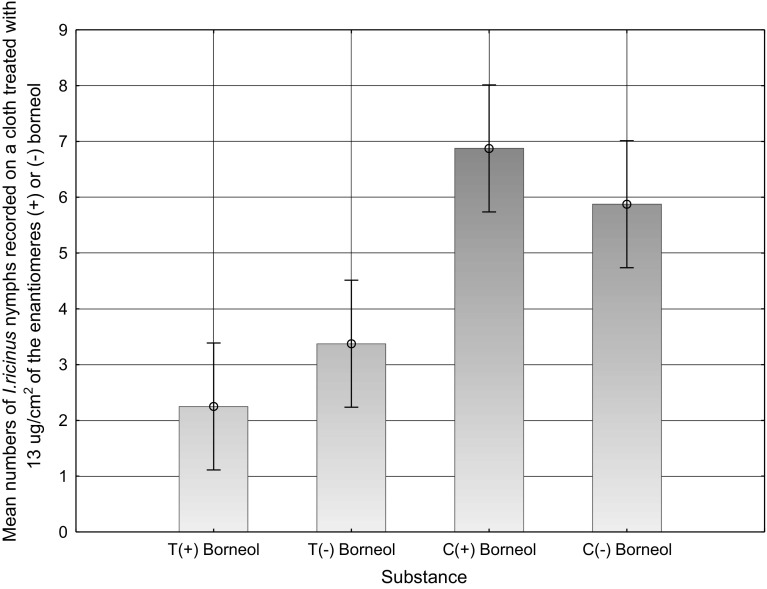
Mean numbers of *Ixodes ricinus* nymphs recorded on a cloth treated with 13 µg/cm^2^ of the enantiomers (+) or (−) borneol compared with its corresponding negative control cloth. Tests were performed in the laboratory

The repellency of each fraction of the turpentine distillation residue against *I. ricinus* nymphs, tested in the lab, is shown in Table 1. Strongest repellency was exhibited by the medium polar fraction 64–84:56–78 R3:2 and borneol (Fluka). The two methods of application gave different results. When the test samples were exchanged after each test (*) a higher repellent effect was found. The more realistic application, once for each test series (**), resulted in a lower repellency value compared to the tests where the sample was exchanged for each replicate, but gave information of long lasting repellent effects, i.e. how suitable the compounds might be in the field. The repellency of the two enantiomers of borneol differed from the control (K–S Two-Sample Test, *p* < 0.01) but not from each other (K–S Two-Sample Test, *p* > 0.1) (Fig. 2).

**Table 1:** Behavioral response (expressed as number attached to cloth after 5 min) of *Ixodes ricinus* nymphs towards fractions of sulfate turpentine at various concentrations, borneol, α-terpineol or the solvent acetone (unless other is written), tested against untreated controls

Fractions and pure substances	N	% attracted nymphs (absolute numbers)		*p*	% repellency/strong repellency
		Test	Control		
R2:1 10% 130 µg/cm^2^*	20	0 (0)	65 (13)	<0.001	100/76.9
R2:1 10% 130 µg/cm^2^**	10	20 (2)	70 (7)	<0.01	85.7/28.5
R2:1 1% 13 µg/cm^2^**	10	60 (6)	100 (10)	NS	40.0/40.0
R2:2 10% 130 µg/cm^2^*	20	0 (0)	80 (16)	<0.01	100/75.0
R2:2 10% 130 µg/cm^2^**	10	0 (0)	50 (5)	<0.05	100/20.0
R2:2 1% 13 µg/cm^2^**	10	40 (4)	50 (5)	NS	20/0
R2:3 10% 130 µg/cm^2^*	20	0 (0)	55 (11)	<0.01	100/82.0
R2:3 10% 130 µg/cm^2^**	10	0 (0)	60 (6)	<0.02	100/16.6
R2:3 1% 13 µg/cm^2^**	10	10 (1)	70 (7)	<0.05	85.7/42.8
R3:1 10% 130 µg/cm^2^*	20	0 (0)	70 (14)	<0.001	100/82.3
R3:1 10% 130 µg/cm^2^**	10	0 (0)	100 (10)	<0.01	100/60.0
R3:1 1% 13 µg/cm^2^**	10	30 (3)	90 (9)	<0.005	66.6/22.2
R3:2 10% 130 µg/cm^2^*	20	0 (0)	70 (14)	<0.001	100/50.0
R3:2 10% 130 µg/cm^2^**	10	0 (0)	100 (10)	<0.01	100/90.0
R3:2 1% 13 µg/cm^2^**	10	20 (2)	100 (10)	<0.02	80.0/60.0
R3:3 10% 130 µg/cm^2^*	20	0 (0)	80 (16)	<0.001	100/87.5
R3:3 10% 130 µg/cm^2^**	10	10 (1)	90 (9)	0.01	88.9/22.2
R3:3 1% 13 µg/cm^2^**	10	70 (7)	90 (9)	NS	22.2/0
α-Terpineol 10% 130 µg/cm^2^**	10	0 (0)	100 (10)	<0.01	100/70.0
α-Terpineol 5% 65 µg/cm^2^**	10	40 (4)	80 (8)	NS	50.0/25.0
α-Terpineol 1% 13 µg/cm^2^**	10	60 (6)	90 (9)	NS	33.3/22.2
Terpinen-4-ol 5% 65 µg/cm^2^**	10	20 (2)	60 (6)	NS	66.6/33.3
Borneol 10% 130 µg/cm^2^**	10	0 (0)	70 (7)	0.01	100/85.7
Borneol 5% 65 µg/cm^2^**	10	0 (0)	100 (10)	0.01	100/80.0
Borneol 1% 13 µg/cm^2^**	10	20 (2)	90 (9)	0.05	77.7/66.6
Borneol 0.1% 1.3 µg/cm^2^**	40	37.5 (15)	62.0 (25)	NS	40.0/na
(+)-Borneol 0.1% 1.3 µg/cm^2^**	20	50.0 (10)	75.0 (15)	NS	33.3/5.0
(−)-Borneol 0.1% 1.3 µg/cm^2^**	20	73.3(11)	75.0 (15)	NS	26.6/0
(+)(−)-Borneol 0.1% 1.3 µg/cm^2^ Mix 100 µl**	20	60.0 (12)	85.0 (17)	NS	29.0/5.8
(+)(−)-Borneol 0.1% 2.6 µg/cm^2^ Mix 200 µl**	20	45.0 (9)	75.0 (15)	NS	40.0/6.0
(+)-Borneol in propanediol 1% 13 µg/cm^2^**	80	22.5 (18)	68.8 (55)	<0.001	67.0/38.2
(−)-Borneol in propanediol 1% 13 µg/cm^2^**	80	26.3 (21)	58.8 (47)	<0.001	55.0/27.6
(+)-Borneol 1% 3 µg/cm^2^ 100 µl**	40	52.5 (21)	75.0 (30)	<0.05	30.0/na
(−)-Borneol 1% 13 µg/cm^2^ 100 µl**	40	50.0 (20)	85.0 (34)	<0.01	41.0/5.9

* 100 µl were applied to a new cloth for each replicate

** 100 µl were applied to one cloth, used for 10 replicates. *N* number of replicates (1 nymph per replicate); *P* probability that frequencies in control and test are similar based on the McNemar’s change test. Ticks that were below the 3 cm level of a 11.5 cm tall test tube (a Falcon™ vial) were regarded as “strongly repelled”

### Field repellency trials

#### Field trial I

**Fig. 3:**
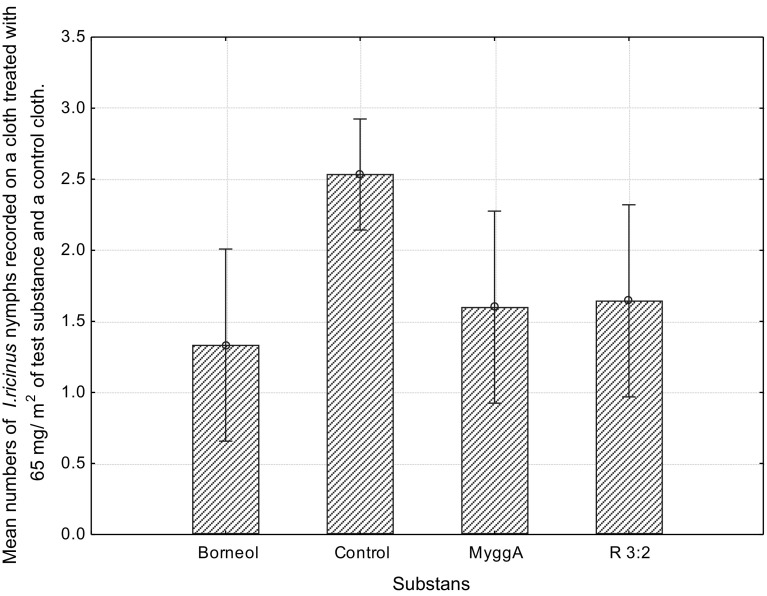
Mean numbers of *Ixodes ricinus* nymphs recorded on a cloth treated with 65 mg/m^2^ of the tick repellent fraction R 3:2, MyggA^®^ (containing 20% DEET), racemic borneol and on a control cloth during 3 days. *Vertical bars* denote 0.95 confidence intervals

Borneol exhibited the highest repellency (51%) against *I. ricinus* nymphs, followed by MyggA^®^ (39%) and the turpentine fraction R3:2 (19%). The Kruskal–Wallis test showed a significant difference [H (3, n = 270) =17.64, *p* = 0.005] between the mean number of nymphs on cloths treated with the test substances, at a concentration of 65 mg/m^2^, compared to the control (Fig. 3). Borneol was statistically different from the control.

#### Field trial II

**Fig. 4:**
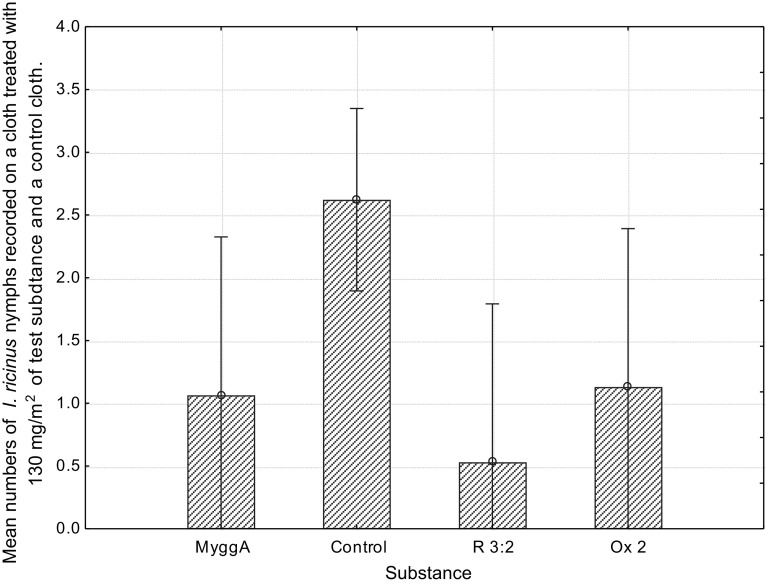
Mean numbers of *Ixodes ricinus* nymphs recorded on a cloth treated with 130 mg/m^2^ of the tick repellent fraction (64–84:56–78) R 3:2, MyggA^®^ (containing 20% DEET), fraction Ox 2 and on a control cloth during 1 day. *Vertical bars* denote 0.95 confidence intervals

The Kruskal–Wallis test showed a significant difference [H (3, n = 90) =17.64, *p* = 0.024] between the mean number of nymphs on a cloth treated with Fraction (64–84: 56–78) R 3:2, oxid (13–21) Ox 2 and the commercially available repellent MyggA^®^ at a concentration of 130 mg/m^2^ when compared to the control cloth (Fig. 4). The field trial was performed over one day.

### Chemical analysis of the most tick repellent turpentine fraction

After repeated separations using MPLC we obtained a fraction (64–84; 56–78) R3:2 with strong tick repellent properties (Table 1). The major part of the fraction (85%) contained monoterpene alcohols; α-dihydroterpineol, α-terpineol, borneol, a monoterpene acetate, thymol, p-cymen-8-ol, myrtenol and cis-verbenol. In addition small amounts of sesquiterpene alcohols were found.

### Chemical analysis of the most active oxidized fractions

Before oxidation this fraction mainly contained (+)- and (−)-α-pinene (−)-β-pinene (+)-3-carene, (−)-β-phellandrene and (+) and (−)-limonene. After oxidation with SeO_2_ and fractionation by MPLC, three fractions (Ox 1–3) were collected guided by TLC, and their tick repellent properties were tested in the laboratory as described above. Fraction (Ox1) 4–8 contained two major components: 2-pinene-10-al (myrtenal, 6,6-dimethyl-2-norpinene-2-carboxaldehyde, 85%) and bicyclo (2,2,1) heptan-3-one-6,6-dimethyl, 2-methylene (15%). Fraction (Ox2) 13–21, which was the most active, consisted of two compounds; 2-pinen-10-ol (myrtenol, 83%) and thymol (17%).

## Discussion

A large number of oxygenated monoterpene hydrocarbons were found in fractions with high tick repellent properties. The repellency of the most active fractions and the pure compounds at a dilution of 10% was comparable to DEET (at a dilution of 20%) during the time the tests were performed. Our results show that only half the amount of the most active fraction is needed to obtain the same repellency as DEET in combination with the additional compounds in MyggA^®.^

One of the main compounds in the most active fraction was borneol. When testing the pure enantiomers separately and in combination they showed medium tick repellency. There was no difference in the repellency between the enantiomers (*p* > 0.1). The commercially available borneol from Fluka contained 20% isoborneol and showed stronger tick repellency than the racemic borneol or the enantiomers separately. The odor of the tick host (humans) is possibly masked by the strongly smelling borneol, as well as by many other known repellents (Pålsson et al. 2008; Garboui et al. 2006; Jaenson et al. 2006a, b), causing disorientation in the ticks. Certain strong smelling compounds (including borneol and other monoterpene alcohols) are also used commercially for masking malodors in human environments. DEET is known to block some of the antennal receptors in mosquitoes (Syed and Leal 2008), which might be an important function also in ticks.

From SeO_2_-oxidation of the fraction containing monoterpene hydrocarbons, we obtained a number of oxygenated monoterpenes resulting from the allylic oxidation of mainly α- and β-pinene, i.e. myrtenal, myrtenol, thymol, borneol, enantiomers of α-terpineol and terpinen-4-ol. These are also present in the highly tick repellent essential oil from *Tanacetum vulgare* (Pålsson et al. 2008) and several essential oils from Egypt (El-Seedi et al. 2012). While most of the oxygenated compounds differed between the most repellent fractions of R3:2 and Ox2, thymol and myrtenol were found in both. A repellent effect based on the mixtures of identified compounds should be further evaluated.

It would be interesting to test the tick repellency effect of the fractions during a longer period. At weaker concentrations, when tested in the laboratory, the tick repellency of fractions started to decline after 45 min. In the field trials the repellency on the third day was much lower than on the first day. However, if a polar less volatile solvent as propylene glycol was used to dissolve the test compounds, a higher percentage of strong repellency was observed, which might depend on the interaction between the compound and the solvent forming a better dispenser than the commonly used volatile acetone (Table 1).

The large amounts of monoterpene hydrocarbons in turpentine are excellent source for producing oxygenated monoterpenes and thus obtain fractions with high tick repellency. Sesquiterpene hydrocarbons present in minor amounts in turpentine, and corresponding oxides also have high tick repellent effect. In an earlier study, we performed tick repellency tests on β-caryophyllene and humulene from *Hyptis suaveolens* (L.) Poit. plants, and synthesized the corresponding oxides and sulfoxides with promising tick repellent results (Ashitani et al. 2015). Oxygenated sesquiterpenes are good candidates for tick repellency; they normally smell good to humans and have lower volatility than the monoterpenes. An inventory focused of sesquiterpenes of different conifer species might result in new lead compounds for new tick repellents.
